# Basal-like subtype of esophageal adenocarcinoma and it’s morphological, molecular and clinical characteristics

**DOI:** 10.1038/s41598-025-08721-9

**Published:** 2025-07-09

**Authors:** Su Ir Lyu, Caroline Fretter, Axel M. Hillmer, Bastian Grothey, Sascha Hoppe, Adrian Georg Simon, Thomas Zander, Karl Knipper, Wolfgang Schroeder, Christiane J. Bruns, Alexander Quaas

**Affiliations:** 1 Faculty of Medicine and University Hospital of Cologne, Institute of Pathology, University of Cologne, Cologne, Germany; 2 Department of General, Visceral and Cancer Surgery, Faculty of Medicine and University Hospital of Cologne, , University of Cologne, Cologne, Germany; 3 Department of Internal Medicine I, University Hospital Cologne, Medical Faculty, University of Cologne, Cologne, Germany

**Keywords:** Esophageal adenocarcinoma, Cytokeratin 5, Cytokeratin 6, Basal-like subtype, Poor prognosis, Cancer therapy, Oesophageal cancer

## Abstract

Esophageal adenocarcinoma (EAC) and squamous cell carcinoma are the two main tumor types of the esophagus, each with distinct morphological and molecular features. While mixed adeno-squamous carcinomas are recognized, it remains unclear whether a basal-like subtype—known from breast and pancreatic adenocarcinomas—also exists in EAC. This study analyzed tumor samples from 953 patients with operable EAC for expression of the basal cytokeratins CK5 and CK6. Tumors with any squamous differentiation were excluded. A small subset (3.4%) showed a basal-like phenotype, characterized by high CK5 and/or CK6 expression and small-luminal tubular growth. High CK5 expression was associated with significantly worse overall survival, and within the neoadjuvantly treated subgroup, it emerged as an independent negative prognostic marker. Tumors with concurrent high CK5 and CK6 expression also showed reduced survival. Exploratory analyses revealed potential associations between CK6 expression and Claudin18.2 status, and between CK5 expression and Y-chromosome loss. These findings indicate the presence of a distinct basal-like subtype within EAC, linked to poor prognosis and possible molecular differences. CK5, alone or in combination with CK6, may serve as a practical biomarker for identifying this subtype and could support the development of more personalized treatment strategies.

**Supplementary Information:**

The online version contains supplementary material available at 10.1038/s41598-025-08721-9.

## Introduction

Esophageal carcinoma is a malignancy with the seventh highest incidence and sixth highest mortality among cancers worldwide^1^. It is often diagnosed late in the disease course due to late clinical symptom presentation, when metastasis has already occurred and therefore is associated with poor 5-year overall survival of around 20%^2,3^.

The two main histological subtypes of esophageal carcinoma are squamous cell carcinoma (ESCC) and adenocarcinoma (EAC). While globally, ESCC is more frequent, two thirds of esophageal carcinomas in high income countries are EAC and most likely associated with the key risk factors of excess body weight, diets high in fat, gastroesophageal reflux disease, and Barrett´s esophagus^1,4,5^. Incidence of EAC has been increasing over the past decade and despite improvements in therapy, long term survival remains poor, particularly among men^6^. Treatment options for esophageal carcinoma consist of surgery, chemo-radiotherapy and newer targeted drug therapies, such as trastuzumab (-deruxtecan) for Her2-positive tumors^7^. ESCC and EAC differ in their morphological characteristics and show distinct pattern on a molecular level^8^. A therapeutically relevant HER2/neu amplification/overexpression is a characteristic feature of EAC, found in approximately one in five cases. Additionally, genomic microsatellite instability (MSI), which is associated with an increased likelihood of response to PD-1/PD-L1-directed immune checkpoint inhibitors, is observed in about 1–3% of EAC cases. Both of these molecular alterations are rarely found in ESCC. According to the results of the CROSS trial, ESCC patients particularly benefit from combined radio-chemotherapy^9^. This neoadjuvant regimen is now a standard treatment for operable ESCC. In contrast, the ESOPEC trial demonstrated that perioperative chemotherapy using the FLOT regimen provides an advantage over neoadjuvant radio-chemotherapy (CROSS) in EAC^10,11^.

Among other cancer entities, a novel basal-like subtype distinct from squamous cell carcinoma (SCC) or classic adenocarcinoma (AC) has been identified and was thus far described in bladder, breast, and pancreatic cancers^12–14^. This basal-like subtype has been associated with worse prognosis and different therapeutic sensitivities compared to classic adenocarcinoma, such as a lack of response to mFOLFIRINOX in basal-like pancreatic ductal adenocarcinoma (PDAC) and insensitivity to hormone blockade in basal-like breast cancer^12–16^. In invasive breast cancer, a panel of antibodies, including cytokeratins 5 and 6, has been established to identify the basal-like subtype^17,18^. In PDAC, cytokeratin 6 was found to be an effective marker for basal-like carcinoma and showed association with worse survival^13^. Cytokeratins are intermediate filament proteins of epithelia and exhibit specific patterns depending on the type of epithelium^19^. In esophageal carcinoma, CK 5/6 routinely stains positively in SCC while AC would generally not stain with these cytokeratins.

Recent findings from our research group (Hoppe et al., manuscript submitted) indicate that upregulation of basal cytokeratins, including CK6 and CK16, is a characteristic feature of EAC tumors that relapse following radiochemotherapy. Using a mouse xenograft model mimicking the standard CROSS treatment protocol, this study identified a therapy-induced basal-like phenotype that correlates with treatment resistance. These results suggest that basal-like characteristics may emerge as an adaptive stress response to therapy, potentially contributing to treatment failure.

In this study, we postulate that a basal-like subtype exists among EAC that exhibits histomorphological criteria definitive of adenocarcinoma while simultaneously showing basal-like protein expressions specific to SCC. Building on the results of Hoppe et al., our study aims to determine whether a pre-existing basal-like EAC subtype can be identified based on CK5 and CK6 expression and whether this subtype is associated with prognosis and therapeutic response. To investigate the presence of a basal-like subtype in EAC, this study aims to characterize tumors using CK5 and CK6 immunohistochemical staining. Additionally, we seek to clarify the prognostic value of this specific subtype in relation to overall survival and clinicopathological characteristics.

## Materials and methods

### Patients and tumor samples

We included a total of 953 patients with EAC that underwent Ivor-Lewis esophagectomy with curative intention between 1998 and 2019 at the University Hospital of Cologne. Data was collected prospectively and analyzed in a retrospective manner. We conducted the pathological evaluation of the specimen according to the 7th edition of the Union for International Cancer Control. Informed consent was obtained in written form from every patient. The local ethics committees approved the study (ethics committee number: 21-1146) and it was conducted in accordance with the declaration of Helsinki. Tissue microarrays (TMAs) were generated in accordance with previously described methods with 1.2 mm tissue cylinders that were punched out with a semiautomated precision instruments and were embedded in empty recipient paraffin blocks^20^. TMAs were constructed using formalin-fixed, paraffin-embedded surgical resection specimens. For each case, one representative tissue core was punched and included in the TMA. Four-micrometer sections of these TMA blocks were transferred to a slide system for further staining.

### Immunohistochemistry

Immunohistochemical stainings were conducted to assess expression of CK5 and CK6. Both antibodies were used according to the manufacturer’s instructions (for details refer to Supplementary Table 1). Stainings were conducted with the automatic staining system Leica BOND-MAX with Leica Bond Polymer Refine Detection Kut (Leica Biosystems, Wetzlar, Germany).

Analysis of the specimens was performed by two pathologists (A.Q. and S. L.). CK5 and CK6 stainings were classified according to their percentage of stained tumor cells (0% = negative, > 0–49% = low positive, > =50% high positive) without considering staining intensity. This approach was chosen for its reproducibility and because staining typically showed a clear-cut positive or negative pattern.

If a specimen showed a high positive staining result for CK5 and/or CK6 in the TMA, we repeated the staining on the whole tumor block to ensure the integrity of the results. This step was included to account for potential sampling bias and to assess the representativeness of TMA-based classification in the context of possible intratumoral heterogeneity.

**Fig. 1:**
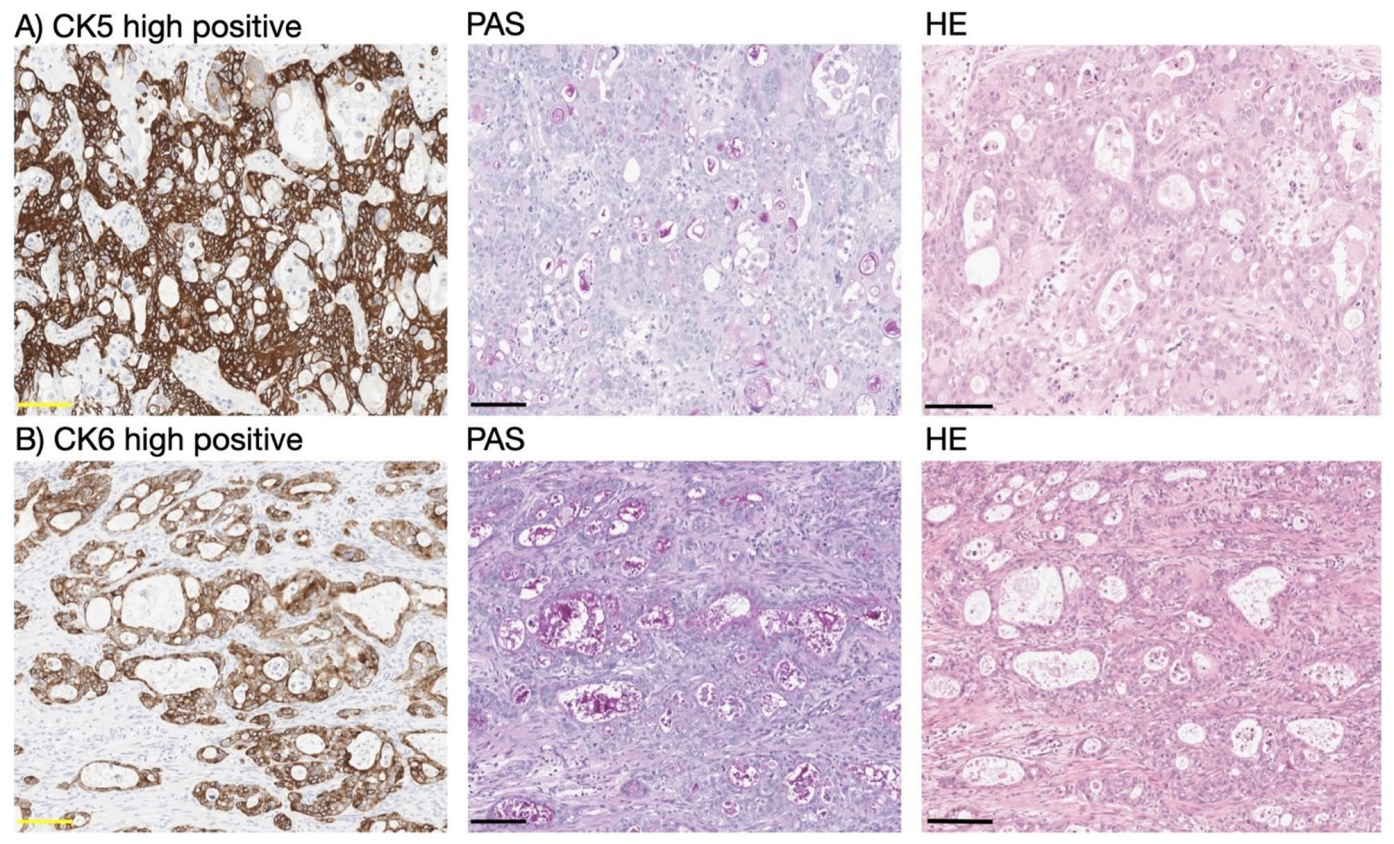
Esophageal adenocarcinoma of tubular type. (**a**) left: high positive CK5 expression, middle: corresponding site with a positive PAS staining, right: corresponding site with a HE staining. (**b**) left: high positive CK6 expression, middle: corresponding site with a positive PAS staining, right: corresponding site with a HE staining. Scale bar: 100 μm. PAS Periodic acid–Schiff.

The basal-like subtype of EAC was defined as follows: tubular or cribriform glandular growth pattern with evidence of Periodic acid–Schiff (PAS)-positive tumor cell-associated mucins. Immunohistochemically, at least one of the two basal cytokeratins 5 or cytokeratins 6 must be detectable in more than 50% of the tumor cells in the whole tumor sections. No squamous cell differentiation must be detectable by standard morphology. Accordingly, all adeno-squamous differentiated esophageal carcinomas were excluded from further analyses. Figure 1 demonstrates samples of CK5 and CK6 high positive tubular adenocarcinoma showing positive PAS staining.

In the present study, we performed secondary correlation analyses between CK5/CK6 expression levels (categorized as negative, low, or high) and a set of molecular markers to explore potential associations between basal-like differentiation and specific molecular features. Immunohistochemical data for SMARCA4, SMARCA2, MLH1, and Claudin18.2 were obtained from previous studies conducted within the same EAC cohort by our group. Antibody details are summarized in Supplementary Table S1. Likewise, data on HER2/neu amplification and Y-chromosome loss were derived from earlier investigations using fluorescence in situ hybridization (FISH). HER2/neu status was assessed using the ZytoLight^®^ SPEC ERBB2/CEN17 Dual Probe Kit (Zytomed Systems GmbH, Germany). Y-chromosome loss was evaluated using the CEP Y (DYZ1) Satellite III DNA SpectrumGreen probe targeting the long arm (Yq11.1–11.23) of the Y chromosome (Abbott GmbH, Wiesbaden, Germany). The evaluation criteria can be found in the respective original publications from which the data were derived^21–24^.

### Statistical analysis

Specimens and follow-up data were collected prospectively and analyzed retrospectively. All statistical analyses were performed using R (version 4.3.0) and RStudio (version 2022.12.0 + 353). Overall survival was defined as time between surgery and death or loss of follow-up. P-values below 0.05 were considered statistically significant. A Chi-square test was conducted to compare qualitative variables. For survival analyses Kaplan–Meier curves were generated and respective log-rank tests were performed. To assess for interdependencies of clinicopathological and survival data, univariate and multivariate Cox regression analyses were conducted. Multivariate Cox regression included only values that exhibited p-values below 0.05 in the univariate Cox regression analysis.

## Results

### Histopathological and clinical characteristics

A total of 953 patients with EAC were included in this study. Of these, 836 patients (87.7%) were male, and 433 patients (45.4%) were older than 65 years. Neoadjuvant therapy was administered in 66.2% of cases, predominantly following either the FLOT regimen or the CROSS protocol. Further information on clinical and pathological characteristics is provided in Table 1.

Among the total study population (*n* = 953), CK5 expression was evaluable in 838 patients and CK6 expression in 814 patients. The discrepancy in evaluable case numbers is due to marker-specific exclusion criteria such as tissue loss, staining artifacts, or insufficient tumor content on the respective TMA cores.

To account for potential spatial heterogeneity, all cases initially classified as CK5 and/or CK6 high based on TMA analysis (*n* = 60) underwent additional whole-slide staining. In 32 of these cases, high expression was confirmed; in the remaining 28 cases, broader tumor areas showed lower expression levels, resulting in reclassification as low. This validation step was incorporated to ensure accurate subgroup assignment. Based on the confirmed classifications, among the 838 evaluable CK5 cases, 813 patients (97.0%) were CK5-negative, 16 patients (1.9%) showed low CK5 expression, and 9 patients (1.1%) showed high CK5 expression. Among the 814 evaluable CK6 cases, 626 patients (76.9%) were CK6-negative, 161 patients (19.8%) showed low CK6 expression, and 27 patients (3.3%) showed high CK6 expression.

Notably, tumors of the basal-like phenotype predominantly displayed compact tubular structures with narrow lumina. These histomorphologic features are illustrated in Fig. 1 and further exemplified in Supplementary Fig. S2, which shows a representative overview H&E section.

### Survival analysis of CK5 and CK6 expression

**Fig. 2:**
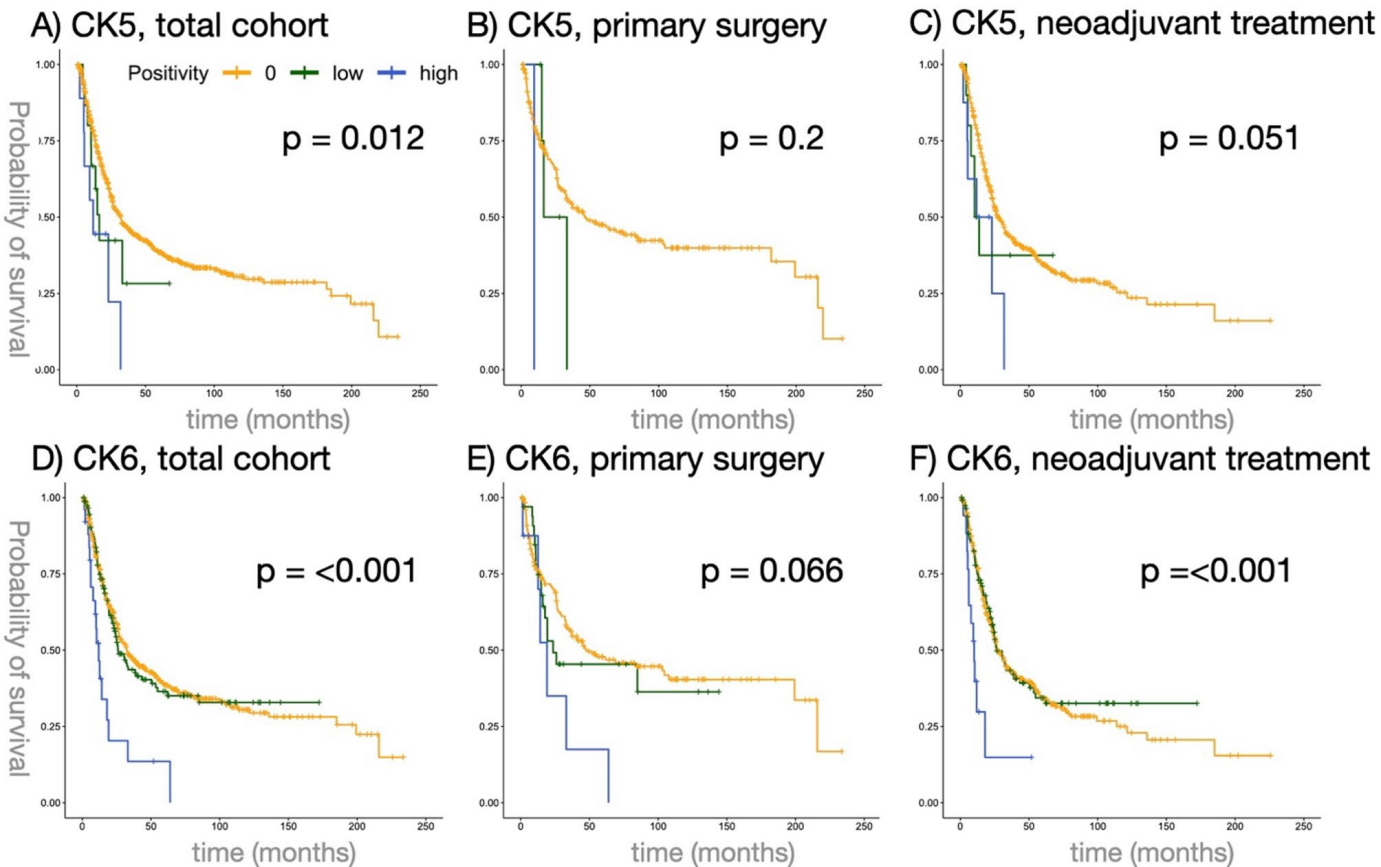
CK5 and CK6 expression are associated with shorter OS in EAC cohort. (**a–c**) OS in patient cohorts of EAC with a negative (orange color), low positive (green color) and high positive (blue color) CK5 expression. (**d–f**) OS in patient cohorts of EAC with a negative (orange color), low positive (green color) and high positive (blue color) CK6 expression. Primary surgery cohort = patients without neoadjuvant treatment before surgery; neoadjuvant treatment cohort = patients treated with CROSS or FLOT regime. A p-value < 0.05 (log-rank test) was considered significant. EAC, esophageal adenocarcinoma; CROSS, Chemo-radiotherapy for oesophageal cancer followed by surgery study; FLOT, fluorouracil-leucovorin-oxaliplatin-docetaxel.

In the total study population, patients with high CK5 or CK6 expression (defined as ≥ 50% of tumor cells positive) showed worse OS than patients with low or negative expression (CK5 median OS for the high positive cohort 11.93 vs. 22.57 months for negative and 13.90 for the low positive cohort, *p* = 0.012, Fig. 2a; CK6 median OS for the high positive cohort 10.12 vs. 23.38 for negative and 21.24 for the low positive cohort, *p* < 0.001, Fig. 2d).

Among neoadjuvantly treated patients with evaluable CK5 expression (*n* = 545), 274 received CROSS, 91 received FLOT, and 180 were treated with other or unspecified protocols (NOS). For CK6-evaluable patients (*n* = 525), 258 underwent CROSS, 82 received FLOT, and 185 received NOS treatment.

In the cohort of patients, who underwent neoadjuvant treatment, worse survival was also observed in the high positive CK6 group (median OS high positive cohort 10.1 vs. 27.6 for negative and 26.3 for low positive cohort, p = < 0.001, Fig. 2f).

No significant differences in OS were observed between CK5 or CK6 expression groups among patients treated with primary surgery (Fig. 2b,e), and no significant difference in OS was found between CK5 expression groups within the neoadjuvantly treated cohort (Fig. 2c).

To assess for potential interdependencies between clinicopathological values and patient outcomes, we performed univariate and multivariate Cox regression analyses.

High CK5 expression in the total cohort was associated with an adverse outcome in the univariate analysis (HR 1.6199, 95% CI 1.162–2.258, *p* = 0.004) and in the multivariate analysis (Table 2). Moreover, within the patient cohort that received neoadjuvant treatment, high positive CK5 expression emerged as an adverse prognostic factor, demonstrating significance in both univariate analysis (HR  1.5575, 95% CI 1.081–2.243, *p* = 0.0173) and multivariate analysis (Table 2).

Even though high positive CK6 expression in the total cohort has shown to be a prognostic factor for worse patient survival in the univariate analysis (HR 1.27115, 95% CI 1.047–1.544, *p* = 0.0155), this finding could not be confirmed in the multivariate analysis (Table 3).

### Combined CK5 and CK6 expression and prognostic impact

**Fig. 3:**
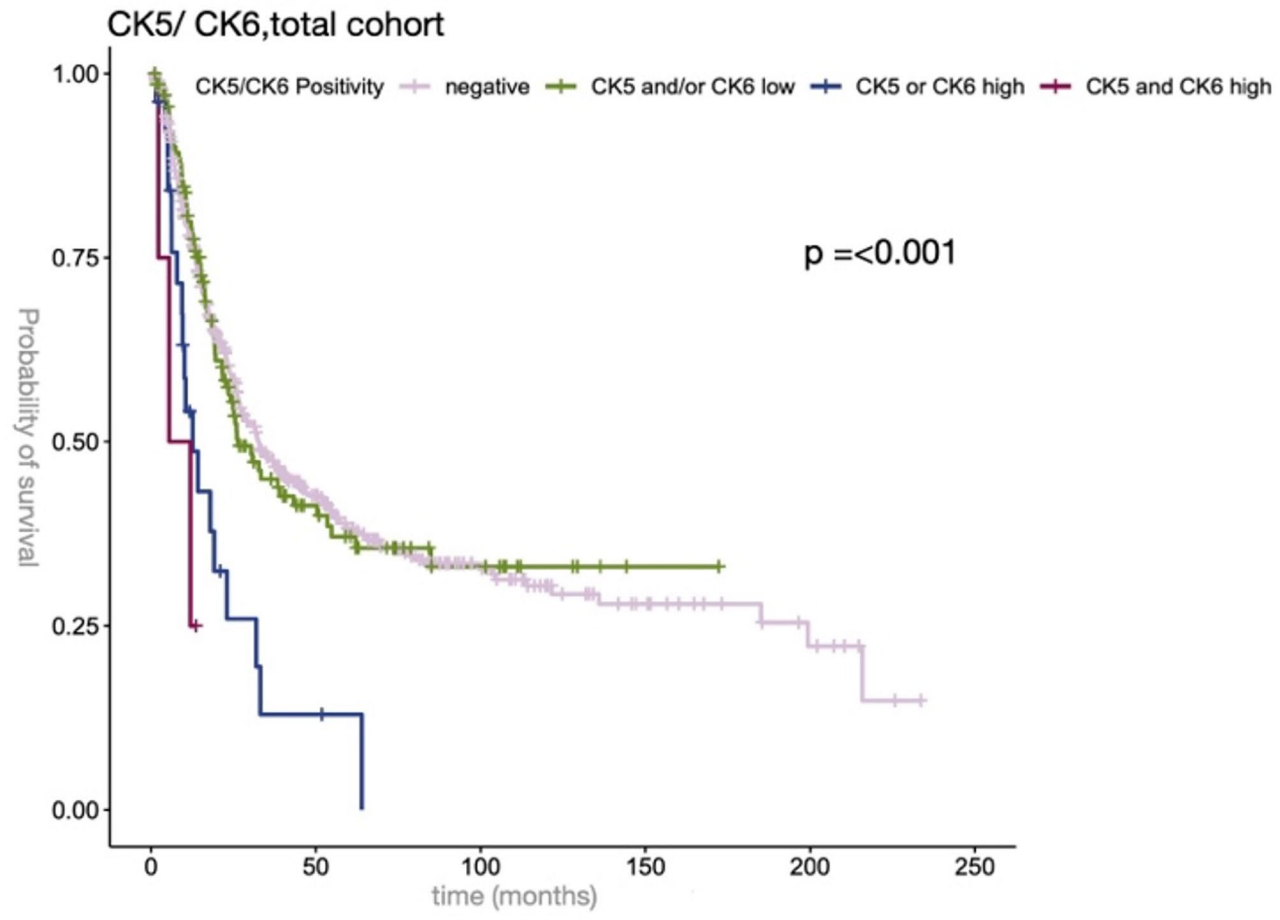
Simultaneous CK5 and CK6 expression in total EAC cohort. OS in patient cohorts of EAC negative for CK5 and CK6 (rose color), low positive for CK5 and/or CK6 (green color), high positive for CK5 or CK6 (blue color), high positive for CK5 and CK6 (purple color). A p-value < 0.05 (log-rank test) was considered significant.

Combined CK5 and CK6 expression data were available for 677 patients. We then performed further statistical analysis based on the combined CK5 and/or CK6 tumor expression. Therefore, we divided the cohort into four groups. Among the examined patients, four showed a simultaneous high positive CK5 and CK6 expression, whereas 26 patients showed either CK5 or CK6 high positivity and 137 patients CK5 and/or CK6 low positivity. 510 patients showed a negative CK5 and CK6 staining. The primary operated and neoadjuvant study populations didn’t show any significant correlation regarding the combined CK5 and CK6 evaluation. Analyzing the total cohort (Fig. 3), high positive simultaneous CK5 and CK6 expression in the total cohort was associated with an adverse outcome in the univariate analysis (HR  1.3004, 95% CI 1.082–1.562, *p* = 0.0051) and in the multivariate analysis (Table 4).

### Correlation of CK5 and CK6 expression with molecular markers

**Fig. 4:**
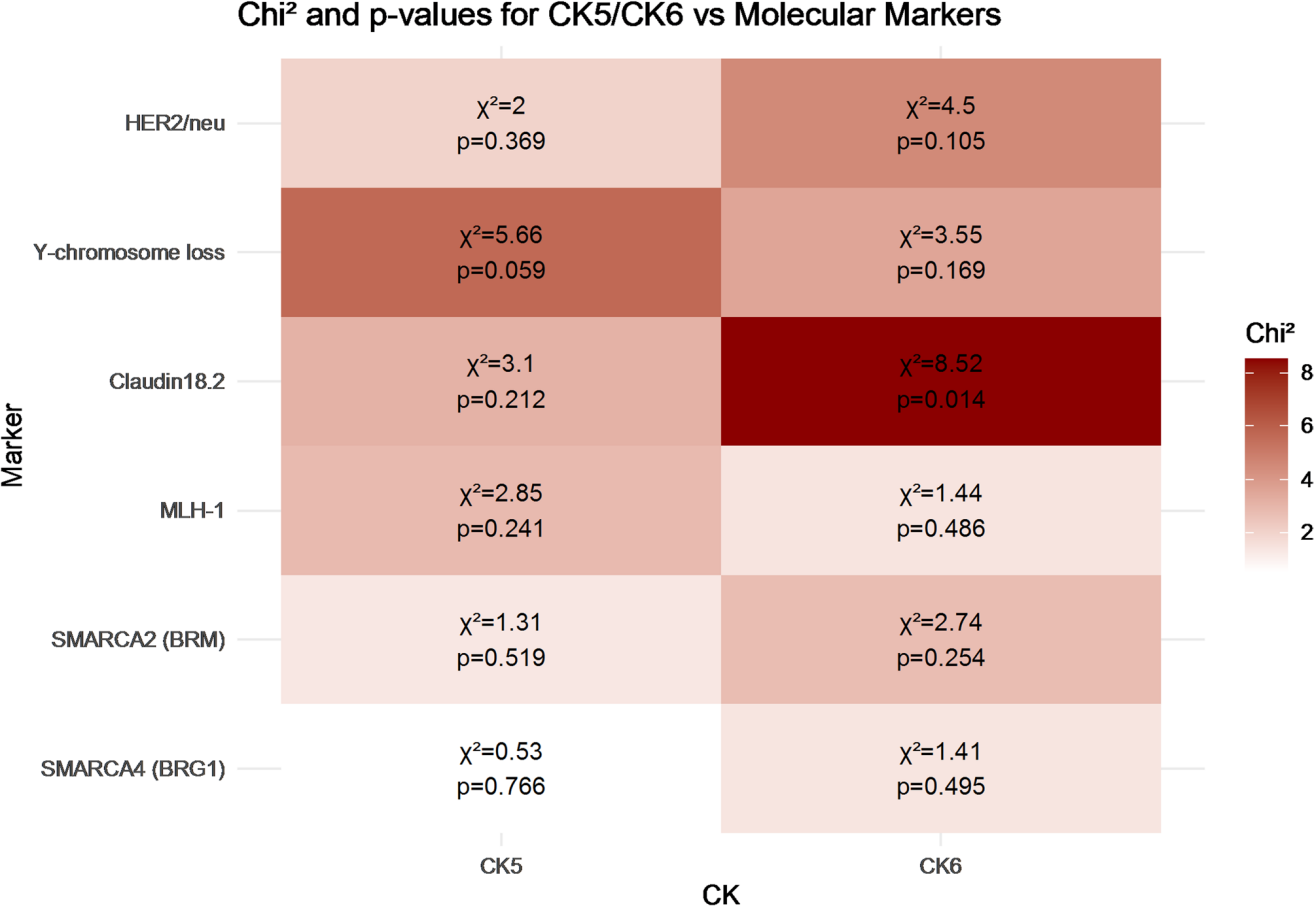
Chi^2^ values and corresponding p-values from Chi-square analyses evaluating the association between CK5 or CK6 expression levels and selected molecular markers (SMARCA4, SMARCA2, MLH-1, Claudin18.2, Y-chromosome loss, HER2/neu). Each tile displays the Chi^2^ statistic (χ^2^) and p-value for the respective marker and cytokeratin. Darker red shading indicates stronger statistical deviation from independence (i.e., higher Chi^2^ values), while lighter shading indicates weaker or no association.

We performed Chi-square analyses to assess the correlation between CK5 expression (categorized as negative, low, or high) and a set of selected molecular markers, including SMARCA4, SMARCA2, mismatch repair-protein MLH-1, Claudin18.2, Y-chromosome loss, and HER2/neu amplification. These markers were chosen based on previous analyses conducted within the same EAC cohort by our group^21–24^. The evaluation criteria applied were consistent with those used in the aforementioned studies. No statistically significant associations were observed (all *p* > 0.05), although a nearly significant trend toward correlation was noted between CK5 expression and Y-chromosome loss (*p* = 0.059) (Fig. 4).

For CK6, a similar analysis was conducted using the same molecular markers. A statistically significant association was observed between CK6 expression and Claudin18.2 status (*p* = 0.0142), while no significant correlations were detected with SMARCA2, SMARCA4, MLH-1, Y-chromosome loss, or HER2/neu amplification (all *p* > 0.1). Tumors with positive Claudin18.2 expression showed a significantly different distribution of CK6 expression levels compared to Claudin-negative tumors. Specifically, CK6-high cases were more frequent among Claudin18.2-negative tumors (3.7% vs. 1.6%, *p* = 0.0142) (Fig. 4).

### Association of CK5 and CK6 expression with clinical data

Among tumors from patients who did not receive neoadjuvant treatment, six cases with CK5 expression showed low expression levels; of these, one was graded G2 and five were G3. High CK5 expression was observed in one case, which was graded G3. Regarding CK6, 45 tumors exhibited low expression, including 25 cases with G2, 19 with G3, and one case without grading information. High CK6 expression was found in ten cases, of which two were graded G2, seven G3, and one had missing grading information. Statistical analysis (Pearson’s Chi-squared test) showed no significant association between histologic grade and the expression levels of CK5 (*p* = 0.58) or CK6 (*p* = 0.53).

In addition to the correlation with grading in the primary surgery cohort, we investigated whether CK5 or CK6 expression influenced patient prognosis differently depending on the type of neoadjuvant therapy received. To assess whether the prognostic effect of CK5 and CK6 expression differed between patients treated with different neoadjuvant protocols, multivariate Cox regression models with interaction terms were performed within the neoadjuvantly treated cohort. No statistically significant interaction was detected for either marker (CK5 × treatment: *p* = 0.96; CK6 × treatment: *p* = 0.70), suggesting that the prognostic impact of high CK5 expression does not vary between FLOT and CROSS protocols. CK6 expression was not prognostically significant in any subgroup. Due to the limited sample size in some of the treatment subgroups, these findings should be interpreted with caution.

In addition to the correlation with grading and treatment response, we also analyzed associations between CK5 and CK6 expression and other clinicopathological parameters (Table 1). Among these, CK5 expression showed statistically significant differences in relation to patient sex (*p* = 0.010) and lymph node status (pN, *p* = 0.009), with high CK5 expression more frequently observed in male patients and in those with nodal metastasis. CK6 expression was significantly associated with patient age (*p* = 0.044), pathological tumor stage (pT, *p* = 0.006), nodal status (pN, *p* < 0.001), lymphatic (*p* < 0.001) and venous invasion (*p* = 0.009).

**Table 1:** General clinicopathological values of the total study population, CK5 and CK6 groups. Bold print marks p-values below 0.05. pT, pathological tumor stage; pN, pathological lymph node status; L, lymphatic invasion; V, venous invasion; Pn, perineural invasion. The bold values represent significant p-values (< 0.05). Absolute numbers are followed by percentages in parentheses. Percentages refer to the respective total number of analyzed cases: the total cohort (*n* = 953), CK5-evaluable cases (*n* = 838), and CK6-evaluable cases (*n* = 814), respectively.

Characteristic	Total	CK5 expression Total *n* = 838 (100)				CK6 expression Total *n* = 814 (100)			
		Negative	Low	High		Negative	Low	High	
	*n* (%)	*n* (%)	*n* (%)	*n* (%)	*p*-value	*n* (%)	*n* (%)	*n* (%)	*p*-value
No. of patients	953 (100)	813 (97.01)	16 (1.91)	9 (1.07)		626 (76.90)	161(19.78)	27(3.32)	
Sex					**0.010**				0.647
Male	836 (87.7)	714 (85.20)	10 (1.19)	8 (0.95)		548 (87.54)	141 (0.17)	22 (0.03)	
Female	117 (12.3)	99 (11.81)	6 (0.72)	1 (0.12)		78 (9.58)	20 (2.46)	5 (0.61)	
Age					0.703				**0.044**
< 65	520 (54.6)	435 (51.91)	8 (0.95)	6 (0.72)		324 (39.80)	101 (12.41)	14 (1.72)	
> 65	433 (45.4)	378 (45.11)	8 (0.95)	3 (0.36)		302 (37.10)	60 (7.37)	13 (1.60)	
Neoadjuvant therapy					0.314				0.082
No	322 (33.8)	286 (34.13)	6 (0.72)	1 (0.12)		234 (28.75)	45 (5.53)	10 (1.23)	
Yes	631 (66.2)	527 (62.89)	10 (1.19)	8 (0.95)		392 (48.16)	116(14.25)	17 (2.09)	
pT					0.191				**0.006**
1	180 (18.9)	151 (18.02)	1 (0.12)	1 (0.12)		122 (14.99)	28 (3.44)	0 (0)	
2	177 (18.6)	150 (17.90)	1 (0.12)	2 (0.24)		104 (12.78)	34 (4.18)	5 (0.61)	
3	565 (59.3)	485 (57.88)	12 (1.43)	5 (0.60)		378 (46.44)	96 (11.79)	18 (2.21)	
4	31 (3.3)	27 (3.22)	2 (0.24)	1 (0.12)		22 (2.70)	3 (0.37)	4 (0.49)	
pN					**0.009**				**< 0.001**
0	391 (41.0)	330 (39.38)	2 (0.24)	2 (0.24)		270 (33.17)	54 (6.63)	3 (0.37)	
1+	561 (58.9)	482 (57.52)	14 (1.67)	7 (0.84)		355 (43.61)	107 (13.15)	24 (2.95)	
x	1 (0.1)	1 (0.12)	0 (0)	0 (0)		1 (0.12)	0 (0)	0 (0)	
L					0.280				**< 0.001**
0	435 (45.6)	364 (43.44)	4 (0.48)	3 (0.36)		287 (35.26)	65 (7.98)	3 (0.37)	
1	355 (37.3)	310 (36.99)	10 (1.19)	5 (0.60)		231 (28.38)	66 (8.11)	22 (2.70)	
x	163 (17.1)	139 (16.59)	2 (0.24)	1 (0.12)		108 (13.27)	30 (3.69)	2 (0.25)	
V					0.589				**0.009**
0	704 (73.9)	598 (71.36)	11 (1.31)	8 (0.95)		465 (57.13)	113 (13.88)	17 (2.09)	
1	91 (9.5)	80 (9.55)	3 (0.36)	0 (0)		56 (6.88)	19 (2.33)	8 (0.98)	
x	158 (16.6)	135 (16.11)	2 (0.24)	1 (0.12)		105 (12.90)	29 (3.56)	2 (0.25)	
Pn					0.501				0.774
0	615 (64.5)	517 (61.69)	11 (1.31)	4 (0.48)		395 (48.53)	102 (12.53)	18 (2.21)	
1	185 (19.4)	166 (19.81)	3 (0.36)	4 (0.48)		129 (15.85)	32 (3.93)	7 (0.86)	
x	153 (16.1)	130 (15.51)	2 (0.24)	1 (0.12)		102 (12.53)	27 (3.32)	2 (0.25)	

**Table 2:** Multivariate analyses for CK5 expression. CI, confidence interval; HR, hazard ratio; pT, pathological tumor stage; pN, pathological lymph node status; G, grading. The bold values represent significant p-values (< 0.05).

Covariate	CK5	
	HR (95% CI)	*p* - value
Total cohort		
Age	1.02 (1.01–1.03)	**< 0.001**
Sex	0.67 (0.48–0.92)	**0.014**
pT	1.32 (1.15–1.53)	**< 0.001**
pN	2.55 (2.03–3.21)	**< 0.001**
Neoadjuvant treatment	1.11 (0.88–1.41)	0.372
CK5 expression	1.43 (1.01–2.02)	**0.043**
Primary surgery cohort		
Age	1.04 (1.01–1.06)	**< 0.001**
Sex	0.59 (0.32–1.08)	0.885
pT	1.68 (1.28–2.22)	**< 0.001**
pN	2.68 (1.65–4.35)	**< 0.001**
G	1.14 (0.76–1.70)	0.526
CK5 expression	0.88 (0.37–2.06)	0.763
Neoadjuvant cohort		
Age	1.01 (1.00-1.02)	0.065
Sex	0.71 (0.48–1.04)	0.078
pT	1.10 (0.93–1.31)	0.266
pN	2.36 (1.82–3.07)	**< 0.001**
CK5 expression	1.48 (1.02–2.15)	**0.040**

**Table 3:** Multivariate analyses for CK6 expression. CI, confidence interval; HR, hazard ratio; pT, pathological tumor stage; pN, pathological lymph node status; G, grading. The bold values represent significant p-values (< 0.05).

Covariate	CK6	
	HR (95% CI)	*p* - value
Total cohort		
Age	1.02 (1.01–1.03)	**< 0.001**
Sex	0.71 (0.51–0.98)	**0.035**
pT	1.31 (1.14–1.52)	**< 0.001**
pN	2.51 (1.98–3.17)	**< 0.001**
Neoadjuvant treatment	1.13 (0.90–1.43)	0.296
CK6 expression	1.15 (0.95–1.39)	0.149
Primary surgery cohort		
Age	1.03 (1.02–1.05)	**< 0.001**
Sex	0.61 (0.33–1.11)	0.106
pT	1.53 (1.16–2.03)	**0.003**
pN	2.75 (1.66–4.54)	**< 0.001**
G	1.17 (0.78–1.74)	0.452
CK6 expression	1.14 (0.82–1.60)	0.432
Neoadjuvant cohort		
Age	1.01 (1.00-1.02)	0.070
Sex	0.77 (0.53–1.13)	0.176
pT	1.14 (0.95–1.35)	0.150
pN	2.31 (1.77–3.02)	**< 0.001**
CK6 expression	1.14 (0.90–1.43)	0.281

**Table 4:** Multivariate analyses for CK6 and CK5 high expression (at least one of both cytokeratins are expressed in more than 50% of tumor cells). CI, confidence interval; HR, hazard ratio; pT, pathological tumor stage; pN, pathological lymph node status; G, grading. The bold values represent significant p-values (< 0.05).

Covariate	CK5 and/or CK6, high	
	HR (95% CI)	*p*-value
Total cohort		
Age	1.02 (1.01–1.03)	**< 0.001**
Sex	0.68 (0.49–0.95)	**0.023**
pT	1.35 (1.16–1.58)	**< 0.001**
pN	2.53 (1.99–3.23)	**< 0.001**
Neoadjuvant treatment	1.11 (0.87–1.41)	0.390
CK5/6 expression	1.20 (1.00-1.44)	**0.049**

## Discussion

In this study, we demonstrate for the first time the existence of a basal-like phenotype in EAC. This subtype is associated with a particularly unfavorable prognosis, also in multivariate statistical analysis. To this end, we examined over 900 operable EACs. In analogy to the already known basal phenotype of ductal adenocarcinoma of the pancreas, the tumor cells had to express basal cytokeratins to a significant extent.

We have defined the basal phenotype of EAC as follows: Tubular or cribriform glandular growth pattern with evidence of PAS-positive tumor cell-associated mucins. Immunohistochemically, at least one of the two basal cytokeratins 5 and/or cytokeratins 6 must be detectable in more than 50% of the tumor cells in the TMA as well as on whole tumor section. No squamous cell differentiation must be detectable by standard morphology. We excluded all adeno-squamous differentiated tumors.

A total of 32 patients fulfilled the criteria for the basal-like subtype after whole-slide confirmation. Among the evaluable cases, high CK5 expression was observed in 9 out of 838 cases (1.1%), high CK6 expression in 27 out of 814 cases (3.3%), and simultaneous high CK5 and CK6 expression in four out of 677 cases (0.6%). These frequencies reflect the small but distinct subset of EAC with basal-like differentiation based on high expression of basal cytokeratins.

Interestingly, tumors classified as basal-like based on high CK5 and/or CK6 expression predominantly exhibited a compact tubular growth pattern with narrow glandular lumina on histomorphology (as shown in Supplementary Figure S2).

A potential limitation of our approach is the use of TMA for the evaluation of CK5 and CK6 expression, which may not fully reflect intratumoral heterogeneity. We observed discrepancies when validating cases on whole tumor sections. Among the initially high-scoring cases in the TMA, only 32 could be confirmed as high expressors upon whole-slide staining, while the remaining 28 showed lower or regionally restricted expression and were reclassified accordingly. These findings underscore the importance of whole-slide validation in studies involving spatially heterogeneous markers and highlight a general limitation of TMA-based biomarker analysis in EAC.

In the basic research conducted by our group (Hoppe et al., manuscript submitted), we observed for the first time an upregulation of cytokeratin 6 in a CROSS-like treated xenograft mouse model of AC. We interpreted this as a consequence of radiochemotherapy and a potential escape mechanism. In the present study, we also aimed to determine whether this basal phenotype can already exist primarily and whether it is detectable in other forms of perioperative therapy (such as after FLOT). We demonstrate both aspects here. While our earlier work focused on cytokeratin 6, we have additionally examined cytokeratin 5 in this study, as it has previously been used to define the basal phenotype of ductal pancreatic carcinoma^25^.

CK5 and CK6 are not functionally identical but can both be found in normal squamous epithelium. CK5 predominates in the basal layer and CK6 in the suprabasal layer^26^. Völker et al. examined the role of both cytokeratins in different cancer entities and found that CK6 was more commonly expressed in various ACs while CK5 stained positive in other cancer types like urothelial carcinoma^26^.

For the reasons mentioned above, we believe it is legitimate to present the results of our work separately for the two basal cytokeratins. Cytokeratins do not exhibit 100% tumor-specific expression distributions. For example, squamous cell carcinomas of the lung or hepatocellular carcinomas may have a certain amount of cytokeratin 7, which is otherwise typically detected in ACs. In line with the non-exclusive expression patterns of cytokeratins, basal cytokeratins were detectable at low levels in 18.6% of the EAC cases examined. When a significant proportion of 50% or more of the carcinoma cells produce these cytokeratins we were able to measure a statistically highly relevant tumor-biological effect with an unfavorable prognosis, which also persisted in the multivariate analysis. We have taken this expression threshold into account in our definition of the basal-differentiated phenotype of EAC. EAC typically displays a heterogenic mutational profile that complicates development and application of targeted treatment options^27,28^. By identifying this novel subtype of EAC with distinct molecular characteristics, our findings may inform the development of more personalized therapeutic approaches for patients with basal-differentiated tumors, potentially addressing the current challenges in treating this malignancy.

A therapeutic effect has already been described in the basal phenotype of the pancreas. Retrospective analyses suggest that this subtype benefits less from chemotherapy especially from a treatment with mFOLFIRINOX^25,29,30^. Findings from our group (Hoppe et al., manuscript submitted) suggest that a basal-like phenotype—characterized by upregulation of CK6 and CK16—may emerge in EAC tumors relapsing after chemoradiotherapy. In light of this, we hypothesize that basally differentiated tumor cell clones might be less responsive to neoadjuvant treatment. This is further supported by our observation of significantly poorer survival in patients with high CK6 expression who underwent neoadjuvant therapy. Basal tumor differentiation may represent a surrogate marker of potentially increased (radio-) chemoresistance that is easy to determine morphologically. A highly relevant question in the future will be how tumors with a basal phenotype induce increased therapeutic resistance and how this resistance can be circumvented.

Importantly, our interaction analyses did not reveal significant differences in the prognostic impact of CK5 or CK6 expression between the CROSS and FLOT neoadjuvant treatment protocols, suggesting that the association of basal cytokeratin expression with patient outcome may be independent of the specific therapy administered.

In line with the prognostic relevance of CK6, we also observed statistically significant associations between CK6 expression and several adverse clinicopathological features, including higher pT stage, nodal metastasis, and lymphovascular invasion. Notably, high CK6 expression was more prevalent in patients with advanced local tumor burden and nodal spread. These associations support the interpretation of CK6 as a possible surrogate marker of more aggressive tumor biology, potentially reflecting a phenotype with increased invasive potential or therapy resistance. While the present study was not designed to establish causality, these findings align with our survival data and warrant further functional investigation.

Although rare, the basal-like subtype defined by high CK5 and/or CK6 expression could represent a biologically distinct subgroup of EAC. Its low prevalence in our cohort highlights the need for larger multicenter cohorts to validate the prognostic relevance and to better characterize the clinical behavior of these tumors.

To further dissect the biological basis and potential therapeutic implications of basal cytokeratin expression in EAC, functional studies will be an important next step. While such mechanistic investigations were beyond the scope of the present histopathological and clinical cohort analysis, we strongly believe that integrated approaches—such as proteomic profiling and the establishment of CK5/CK6-modulated organoid or cell line models—will be essential to elucidate the downstream pathways associated with basal cytokeratin expression and their potential role in therapy resistance. Particularly in light of our findings, we aim to pursue such analyses in future studies to better characterize the basal-like subtype of EAC at a functional level.

In addition to the survival-based characterization, we also explored potential molecular associations. In an exploratory analysis, we assessed the potential associations between CK5 and CK6 expression and a panel of molecular markers previously analyzed within the same cohort. While no statistically significant correlations were found for CK5, a nearly significant trend was noted for the tumors with a Y-chromosome loss (*p* = 0.059). We previously showed that almost 60% of EAC exhibit a complete loss of the Y chromosome. We interpreted this loss as an expression of general chromosomal instability. It is remarkable that there is at least an almost significant association with the basal-like phenotype. CK6 expression was significantly associated with Claudin18.2 status (*p* = 0.0142), with CK6-high tumors occurring more frequently among Claudin18.2-negative cases. This observation may indicate partially distinct molecular programs within these subgroups, although further functional or mechanistic studies would be required to explore this in more detail.

Given the prognostic relevance of CK5 and CK6 expression, future efforts could focus on the development of a clinically applicable prognostic nomogram integrating basal cytokeratin expression, TNM staging, and molecular biomarkers. However, before such a tool can be implemented, the limitations highlighted in this study—particularly the need for functional validation and mechanistic characterization—must be addressed. Our current findings serve as a foundational step in defining the basal-like subtype of EAC, and further studies in larger cohorts with integrated multi-omics approaches will be essential to enable personalized risk stratification and guide therapeutic decision-making.

In summary, high expression of CK5—alone or in combination with CK6—may serve as a simple and practical tool to identify the prognostically unfavorable basal-like subtype of EAC. These results may have clinical implications, suggesting that patients with high CK5 or CK6 expression in EAC could be candidates for intensified therapeutic or surveillance approaches.

## Conclusions

The basal-like subtype of EAC exists and serves as an independent risk factor for reduced overall survival in EAC patients, both in primary and neoadjuvant-treated patients. These insights could promote extensive clinical application in tailoring personalized therapy pathways.

## Electronic supplementary material

Below is the link to the electronic supplementary material.

Supplementary Material 1

## Data Availability

The datasets generated and analyzed during the current study are available from the corresponding author on reasonable request.
