# Supplementary material for: Basal-like subtype of esophageal adenocarcinoma and it’s morphological, molecular and clinical characteristics
Source: Sci Rep. 2025 Jul 9;15:24680. doi: 10.1038/s41598-025-08721-9 (PMC12241421; doi:10.1038/s41598-025-08721-9)
Supplement: Supplementary file 1 — Supplementary Material 1 [file 41598_2025_8721_MOESM1_ESM.pdf]

Supplement Information

Supplementary Figure S1. Representative images of performed immunohistochemical stainings (left: negative, middle: low positive, right: high positive): (A) CK5, (B) CK6. Scale bar: 50 µm.

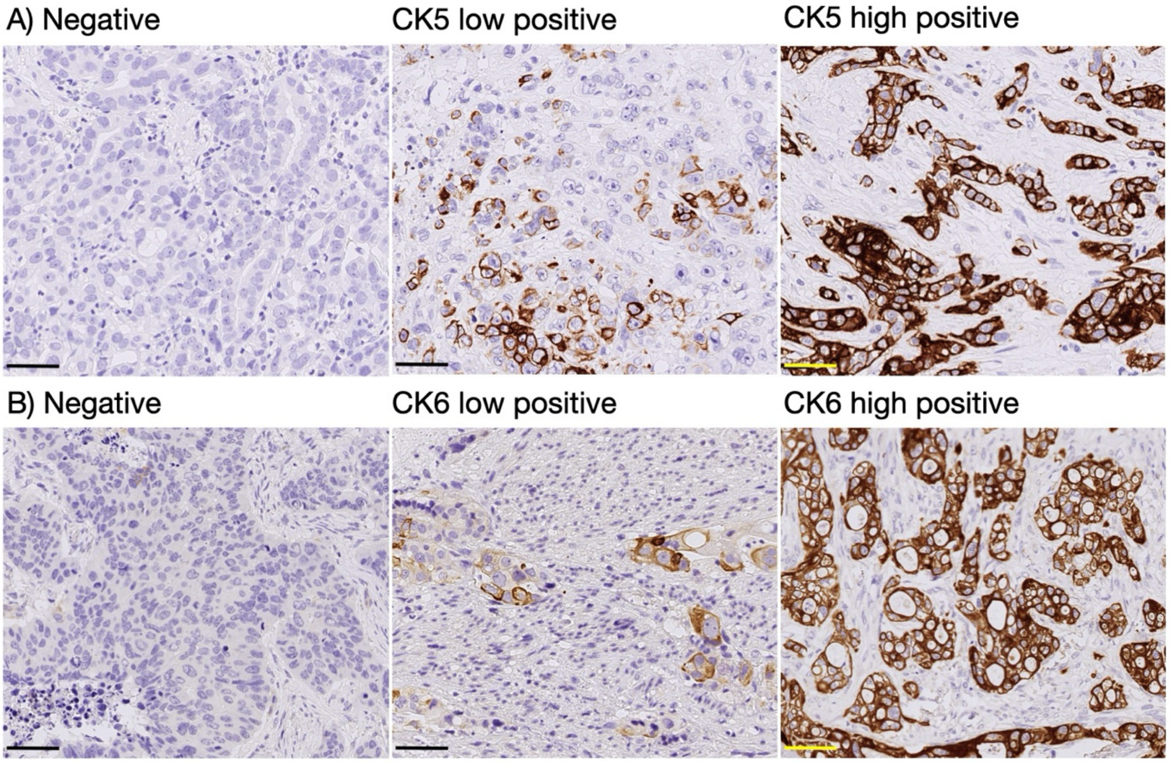

Supplementary Figure S2. Representative H&E image of a surgically resected esophageal adenocarcinoma of the immunohistochemically verified basal-like subtype. The tumor displays compact tubular structures with narrow lumina. Scale bar: 100 µm.

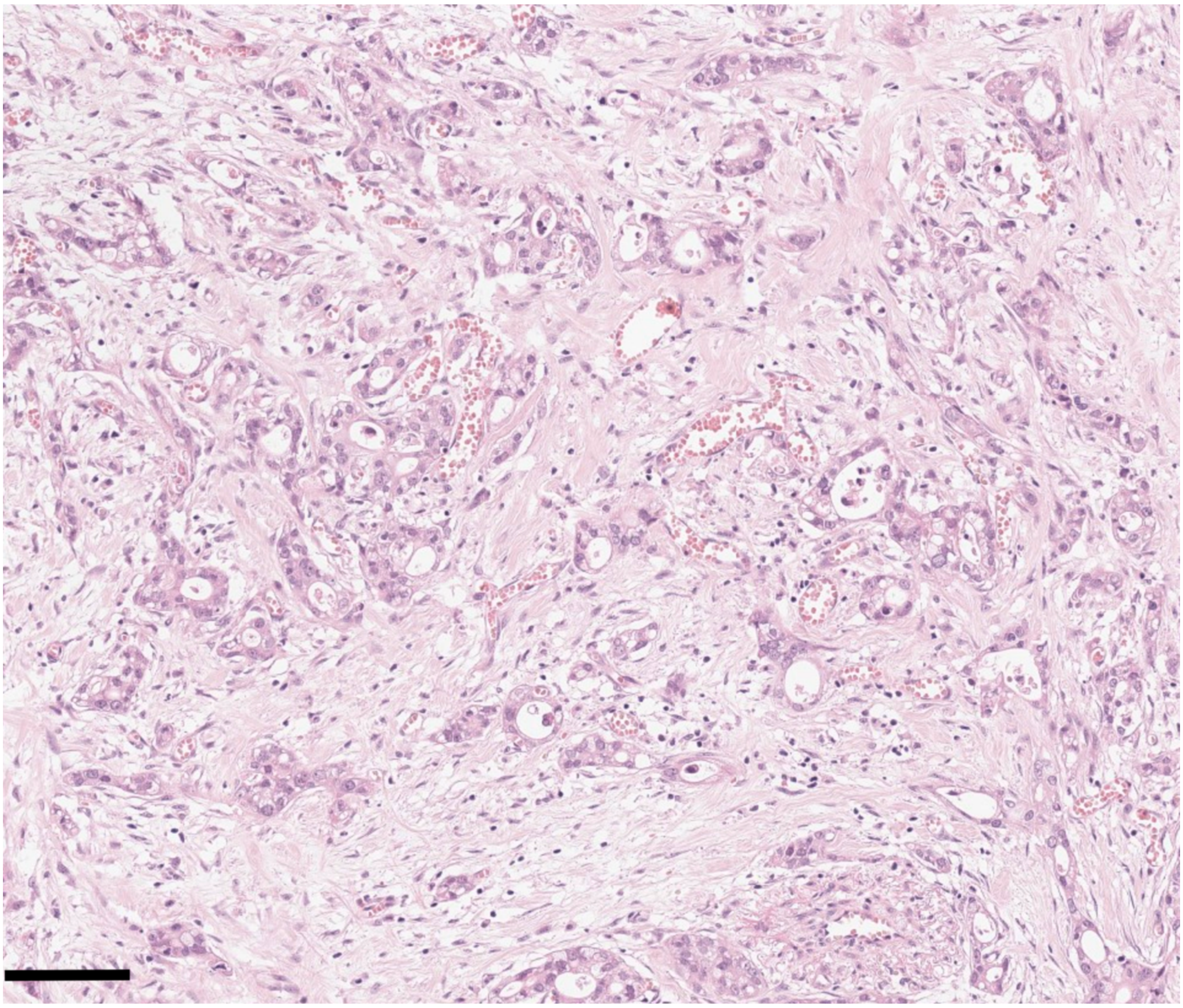

Supplementary Table S1. Detailed antibody information. A Appendix vermiformis, S stomach, T tonsil.

Antibody	Manufacturer	Clone	Dilution	Pretreatment	Control tissue	Order number
CK5	Cell Signaling	E2T4B	1:1000	Citrate	T	#81817
CK6	Huabio	SN71-07	1:200	EDTA	T	ET1611-70
p40	Zytomed	Polyclonal	1:50	Citrate	A/T	ACI3030B
BRG1 (SMARCA4)	Abcam	EPNCIR111A	1:300	EDTA	A	Ab110641
BRM (SMARCA2)	Cell-Signaling	D9E8B	1:50	EDTA	A	11966S
MLH-1	Roche	M1	none	EDTA	A	790-5091
Claudin 18.2	Roche	43-14A	none	EDTA	S	8504148001
